# Coagulation phenotype of wild-type mice on different genetic backgrounds

**DOI:** 10.1177/0023677218811059

**Published:** 2018-11-12

**Authors:** Alexandra Kopić, Karima Benamara, Maria Schuster, Peter Leidenmühler, Alexander Bauer, Helmut Glantschnig, Werner Höllriegl

**Affiliations:** Shire, Vienna, Austria

**Keywords:** thromboelastography, coagulation parameters, reduction, strain differences, genetic background

## Abstract

Genetically engineered mouse models are used to investigate beneficial treatment in haemophilia by comparison with wild-type mice. It has been recognized that wild-type and haemophilic mice of different genetic backgrounds show different bleeding phenotypes. We assessed *ex-vivo* coagulation parameters in nine wild-type substrains of 129S1/Sv, BALB/c and C57BL/6 mice applying thromboelastography (TEG), activated partial thromboplastin time (aPTT), prothrombin time (PT) and fibrinogen levels. The comprehensive *ex-vivo* data are discussed in view of results from a tail-tip bleeding assay. Time to first clot formation (*R*-time) showed higher within-substrain (CV range: 28–54%) and higher between-substrain (median range: 25.53–42.60 min) variation for BALB/c than for C57BL/6 mice (CV range: 14–31%; median range: 22.45–24.93 min). Median R-time for 129S1/Sv mice was 30.42 min (CV: 33%). No distinct strain differences were observed for maximum amplitude (MA), aPTT, or PT, but males generally showed higher MA and shorter aPTT than females. Males of all substrains had higher fibrinogen levels than females. The heightened *in-vivo* variability (CV range: 81–171%; median range: 36.00–469.50 mg) in the tail-tip bleeding assay and increased blood loss in wild-type C57BL/6 male mice was not reflected in *ex-vivo* coagulation parameters. In general, *ex-vivo* coagulation results appeared consistent within substrains, but showed substrain and sex differences of variable magnitudes. We conclude that alignment of the mouse substrain genetic background to the experimental model is critical to reduce data variability and animal numbers.

## Résumé

Les modèles de souris génétiquement modifiées sont utilisés pour étudier les traitements bénéfiques de l'hémophilie par rapport aux souris de type sauvage. Il a été reconnu que les souris de type sauvage et hémophiles d’origines génétiques différentes présentaient différents phénotypes de saignement.

Nous avons évalué les paramètres de coagulation ex vivo de neuf sous-souches de type sauvage de souris 129S1/SV, BALB/c et C57BL/6 en utilisant des tests de thromboélastographie (TEG), de temps de céphaline activée (TCA), de prothrombine (PT) et de niveaux de fibrinogène. Les données complète *ex vivo* sont discutées à la lumière des résultats d'un test de saignement du bout de la queue. Le délai avant la formation du premier caillot (temps-R) présentait des variations plus importantes au sein des sous-souches (plage de CV : 28 – 54 %) et plus élevées entre sous-souches (fourchette médiane : 25,53 - 42,60 min.) de souris BALB/c que de souris C57BL/6 (plage de CV : 14 – 31 %; fourchette médiane : 22,45 - 24,93 min.). Le temps-R médian pour les souris 129S1/Sv était de 30,42 min. (CV : 33 %). Aucune différence de souche distincte n’a été observée quant à l'amplitude maximum (AM), le TCA ou le TP, mais les mâles présentaient généralement une AM plus élevée et des TCA plus courts que les femelles. Les taux de fibrinogène des mâles issus de toutes les sous-souches étaient plus élevés que ceux des femelles. La variabilité accrue *in vivo* (plage de CV : 81 -171 %, fourchette médiane : 36,00 – 469,50 mg) lors du test de saignement du bout de la queue, et une perte de sang accrue chez les souris mâles de types sauvage C57BL/6, n’étaient pas corroborées par les paramètres de coagulation *ex vivo*. Les résultats de la coagulation *ex vivo* semblaient généralement cohérents au sein des sous-souches, mais montraient des différences d’amplitude variées entre sous-souches et sexes. Nous en concluons que l'alignement de l’origine génétique de la sous-souche de souris au modèle expérimental est essentiel pour réduire la variabilité des données et le nombre d'animaux utilisés.

## Abstract (de)

Genetisch veränderte Mausmodelle dienen zur Untersuchung optimaler Behandlungsmöglichkeiten der Hämophilie im Vergleich zu Wildtyp-Mäusen. Es wurde erkannt, dass Wildtyp- und hämophile Mäuse unterschiedlicher genetischer Herkunft unterschiedliche Hämostase-Phänotypen aufweisen. Wir bewerteten *Ex vivo-*Koagulationsparameter in neun Wildtyp-Unterstämmen von 129S1/Sv-, BALB/c- und C57BL/6-Mäusen mittels Thrombelastographie (TEG), aktivierter partieller Thromboplastinzeit (aPTT), Prothrombinzeit (PT) und Fibrinogenspiegel. Die kompletten *Ex vivo*-Daten werden anhand der Ergebnisse eines Schwanzspitzen-Hämostase-Assays diskutiert. Die Zeit bis zur ersten Gerinnselbildung (R-Zeit) zeigte eine höhere Variation innerhalb von Unterstämmen (CV-Bereich: 28 – 54 %) und zwischen Unterstämmen (Median-Bereich: 25,53 - 42,60 min) für BALB/c als bei C57BL/6-Mäusen (CV-Bereich: 14 – 31 %; Median-Bereich: 22,45 - 24,93 min). Die mediane R-Zeit für 129S1/Sv-Mäuse betrug 30,42 min (CV: 33 %). Bezüglich maximaler Amplitude (MA), aPTT bzw. PT wurden keine ausgeprägten Unterschiede zwischen Stämmen beobachtet, aber männliche Tiere wiesen im Allgemeinen eine größere MA und eine kürzere aPTT auf als weibliche. Männliche Tiere aller Unterstämme hatten einen höheren Fibrinogenspiegel als weibliche. Die erhöhte *In vivo*-Variabilität (CV-Bereich: 81 -171 %, Median-Bereich: 36,00 – 469,50 mg) im Schwanzspitzen-Hämostase-Assay und der erhöhte Blutverlust bei männlichen Wildtyp-C57BL/6-Mäusen spiegelte sich nicht in E*x vivo*-Koagulationsparametern wider. Im Allgemeinen waren *Ex vivo-*Koagulationsergebnisse innerhalb von Unterstämmen konsistent, wiesen aber Unterschiede variabler Größen zwischen Unterstämmen und Geschlecht auf. Wir kommen zu dem Schluss, dass die Ausrichtung des genetischen Hintergrunds von Mäuse-Unterstämmen auf das Versuchsmodell entscheidend ist, um Datenvariabilität und Tierverbrauch zu reduzieren.

## Resumen

Los modelos de ratón creados genéticamente se utilizan para investigar el tratamiento beneficioso en la hemofilia comparándolos con los ratones de tipo salvaje. Se ha reconocido que los ratones hemofílicos y los de tipo salvaje con distintos historiales genéticos muestran distintos fenotipos de sangrado. Evaluamos parámetros de coagulación *ex vivo* en nueve subcepas de tipo salvaje de ratones 129S1/Sv, BALB/c y C57BL/6 aplicando tromboelastografia (TEG), tiempo de tromboplastina parcial activado (aPTT), tiempo de protrombina (PT) y niveles de fibrinógeno. Los exhaustivos datos *ex vivo* son discutidos en vistas a los resultados de las pruebas de sangrado en la punta de la cola. El tiempo hasta la primera formación de coágulos (tiempo R) mostró una variación superior dentro de las subcepas (rango CV: 28 - 54%) y una variación superior entre las subcepas (rango medio: 25,53 - 42,60 min) para BALB/c que para los ratones C57BL/6 (rango CV: 14 - 31%; rango medio: 22,45 - 24,93 min). El tiempo R medio para los ratones 129S1/Sv fue 30,42 min (CV: 33%). No se observan unas diferencias de cepas distintivas para la máxima amplitud (MA), aPTT o PT, pero los los machos por lo general mostraron una MA superior y un aPTT inferior que las hembras. Los machos de todas las subcepas tuvieron unos niveles de fibrinógeno superiores que las hembras. La variabilidad *in vivo* intensificada (rango CV: 81 -171%, rango medio: 36,00 – 469,50 mg) en la prueba de sangrado en la punta de la cola y el incremento de pérdida de sangre en los ratones macho de tipo salvaje C57BL/6 no se reflejó en los parámetros de coagulación *ex vivo*. Por lo general, los resultados de coagulación *ex vivo* parecieron consistentes entre las subcepas pero mostraron diferencias entre sexos y subcepas de magnitudes variables. Podemos concluir que la alineación del historial genético de la subcepa del ratón con el modelo experimental es crítico para reducir la variabilidad de datos y los números de animales.

To test the efficacy of therapeutically administered coagulation factors, research groups use various animal models including bleeding and *ex-vivo* assays in haemophilic knock-out and wild-type mouse strains.^1–4^ *Ex-vivo* coagulation parameters are also important endpoints in evaluating the preclinical safety and toxicity of a compound. It is thus necessary to understand the background data of the animals used.

The tail-tip bleeding assay is a widely-used pharmacodynamics model in haemostasis research; however, the literature reports differences in bleeding phenotypes in wild-type mouse strains.^2–6^ Schiviz and colleagues^6^ also demonstrated an influence of genetic background on genetically modified mice with a haemophilia A bleeding phenotype.

Due to the potential additional influence of physiologic and environmental factors in *in-vivo* testing, the present study was conducted to assess whether differences in wild-type mice strains are also observed *ex vivo*. For this purpose, we characterized the coagulation phenotype of four substrains of C57BL/6 and BALB/c mice, respectively, and one substrain of the 129S1/Sv mouse. To this end, we applied thromboelastography (TEG), measurements of coagulation parameters, and also plasma fibrinogen. TEG is a well-accepted method to test patients' coagulation status and has been applied to a variety of preclinical haemophilia models including mice to assess the efficacy of administered clotting factors.^7,8^

Activated partial thromboplastin time (aPTT), prothrombin time (PT) and fibrinogen levels are also indicators of the influence of haemophilia drugs on the coagulation system^9,10^ and are frequently included in the evaluation of preclinical efficacy and safety of experimental therapies.

These parameters were therefore included in the *ex-vivo* coagulation assessments, and the data are presented and discussed in view of results from a previous tail-tip bleeding study in the same substrains.^6^ Here, we demonstrate substrain and sex differences in critical *ex-vivo* coagulation parameters and suggest consideration of these findings in the experimental design of mouse coagulation studies.

## Animals

Substrains of C57BL/6 mice (C57BL/6 JCrl, C57BL/6 NCrl, C57BL/6 BomTac, C57BL/6 OlaHsd) and BALB/c mice (BALB/c AnCrl, BALB/c AnNTac, BALB/c OlaHsd, BALB/c J) and one substrain of 129Sv mice (129S1/Sv mJ) were used. 129S1/Sv mJ, C57BL/6 JCrl, C57BL/6 NCrl, and BALB/c AnCrl mice were purchased from Charles River Inc. (Sulzfeld, Germany); C57BL/6 BomTac and BALB/c AnNTac mice were from Taconic Farms Inc. (Bomholt, Denmark); C57BL/6 OlaHsd, and BALB/c OlaHsd mice were obtained from Harlan Laboratories SRL (San Pietro al Natisone, Italy; and BALB/c J mice were from The Jackson Laboratory (Bar Harbor, ME, USA) via Charles River Inc. (Sulzfeld, Germany). These wild-type mouse strains were chosen as they are of the same genetic background as the FVIII knock-out mice, the principal animal model for haemophilia A, and used in our previous study describing bleeding phenotypes.^6^

Animals were 8–10 weeks of age and housed in type III cages (Tecniplast, Buguggiate, Italy) in groups of five (males or females), and received food (ssniff Spezialdiaeten, Soest, Germany) and water (tap water) *ad libitum*. Nesting material from aspen wood and paper pads was provided to ensure normal behaviour. There was no blinding in the studies described.

Animal facilities were AAALAC accredited (AAALAC accreditation unit no. 001228). All animal experiments followed a protocol authorized by the Austrian Authorities on Animal Experiments and the Institutional Animal Care and Use Committee (IACUC).

## Material and methods

Groups of 10 male (m) and 10 female (f) mice (≥23 g body weight) were anaesthetized via intraperitoneal injection of 100–150 mg/kg ketamine and 10–15 mg/kg xylazine 10 minutes before performing the procedures outlined below. Groups of 10 mice (5m/5f) were randomly assigned to TEG or to coagulation assessments. State of surgical tolerance was assessed by qualified staff by checking the loss of pedal reflexes (firm toe pinch). Mice were humanely killed by cervical dislocation immediately after exsanguination. Experimental procedures were performed during normal working hours in a laboratory room not directly connected to the animal housing rooms.

The exploratory character of these experiments, without knowing effect sizes, prevented mathematical sample size estimation. However, the sample size was based on years of experience working with similar animal models lacking coagulation factor activity and should therefore be sufficient to describe any effects/differences.

## Thromboelastography

The abdominal cavity was opened by a median incision and the *Vena cava caudalis* exposed and punctured. Venipuncture was performed with a 2-ml syringe (25 gauge needle) filled with 0.1 ml sodium citrate; 1 ml of citrated whole blood was drawn (blood:citrate = 10:1); 340 µl citrated blood was then mixed with 20 µl CaCl_2_ and TEG analysis was started immediately using a Heamoscope TEG® 5000 Thrombelastograph® Hemostasis Analyzer (Haemonetics Corp., Braintree, MA, USA). TEG was stopped after all relevant parameters were obtained or cancelled after 3 hours when there was no clot formation. For each animal, two TEG runs were performed in parallel, and the means from both runs were used for statistical analyses. Only TEG curves with results of ± 20% of the *R*-time in the double determination were evaluated to ensure repeatability of measurements. Parameters assessed were *R*-time (min) as the time of latency until initial fibrin formation, and maximum amplitude (MA, mm) as a measurement for the ultimate strength of the fibrin clot.

## Coagulation parameters and fibrinogen

As described above, 1 ml of citrated whole blood was drawn (blood:citrate = 10:1) and platelet poor plasma prepared by centrifugation (2x 10 min at 1100 *g*; Multifuge 1 S-R, Heraeus, Newport Pagnell, UK). Activated partial thromboplastin time (aPTT; s), prothrombin time (PT; s) and fibrinogen (mg/dl) were assessed (ACL Elite Pro, Instrumentation Laboratory, Lexington, MA, USA).

## Statistical analyses

### Descriptive statistics

Between-substrain and within-substrain variation were assessed in a descriptive manner per mouse strain (BALB/c and C57BL/6) for males and females combined. Between-substrain variation was assessed by ranges of medians within each strain and within-substrain variation by ranges of coefficients of variation (CVs).

### Comparison of parameters between the sexes

Differences in parameters between males and females were assessed using median ratios and corresponding nonparametric two-sided 95% confidence intervals (CIs) via R function *pairwiseCI* of R package *pairwiseCI*.^11^ A two-sided 95% CI for the ratio not containing the value 1 is equivalent to rejecting the null hypothesis of no difference against the two-sided alternative at the 5% level of statistical significance. Two-sided 95% CIs were interpreted with caution due to the small sample size per substrain and sex. As these comparisons were considered to be exploratory, no adjustment for multiplicity was applied. All calculations were performed with R version 3.2.2.^12^

## Results

### TEG

**Figure 1.**
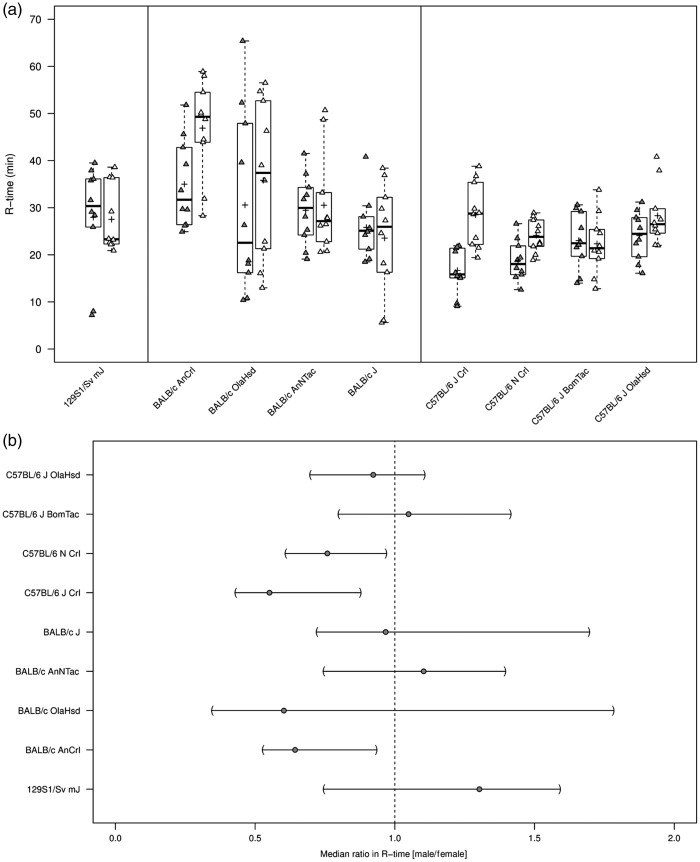
Thromboelastography (*R*-time) in citrated whole blood from wild-type 129S1/Sv mJ, BALB/c, and C57BL/6 substrains. (a) *R*-time (min) summarized graphically by substrain and sex using boxplots (grey = males; white = females). Boxplots: The lower edge of the box represents the 25th percentile (or 1st quartile), the upper edge of the box represents the 75th percentile (or 3rd quartile), and the line within the lower edge and the upper edge of the box indicates the median. The distance from the lower edge to the upper edge of the box represents the inter-quartile range (IQR). A whisker is drawn above the 75th percentile to the largest data value that is less or equal to the value that is 1.5 × IQR above the 75th percentile. A whisker is drawn below the 25th percentile to the smallest data value that is less or equal to the value that is 1.5 × IQR below the 25th percentile. The cross represents the arithmetic mean. Individual measurements were added to the boxplots, where the exact horizontal position of plotting symbols was randomly determined. (b) The median ratio in *R*-time between sexes and corresponding two-sided 95% CIs for each substrain. A two-sided 95% CI for the ratio not containing the value 1 is equivalent to rejecting the null hypothesis of no difference against the two-sided alternative at the 5% level of statistical significance. Two-sided 95% CIs for should be interpreted with caution due to the small sample size per substrain and sex.

All repeated measurements of plasma samples were within 20% *R*-time, and thus *R*-time and MA were assessable in all animals (*n* = 10 per substrain) (Table 1). Median *R*-time was 30.42 min (coefficient of variation, CV 33%) in 129S1/Sv mJ mice. *R*-time showed higher within-substrain (CV range: 28–54%) and higher between-substrain variation (median range: 25.53–42.60 min) for BALB/c in time to first clot formation (*R*-time) compared with C57BL/6 mice (CV range: 14–31%; median range: 22.45–24.93 min) (Figure 1(a)). Sex differences in median ratios (male/female) in *R*-time appeared marked and were significant for particular substrains (C57BL/6 NCrl, C57BL/6 JCrl). However, no clear sex differences in either direction were observed in any other strains (Figure 1(b)).

**Table 1.** Descriptive statistics for *in-vivo* and *ex-vivo* parameters from wild-type 129S1/Sv mJ, BALB/c, and C57BL/6 substrains. Shown are medians and CVs for each parameter and substrain. In addition, the median per sex is presented for each parameter and substrain.

Model	Parameter	Statistic	*N* =	129S1/Sv mJ	BALB/c AnCrl	BALB/c OlaHsd	BALB/c AnNTac	BALB/c J	C57BL/6 J Crl	C57BL/6 N Crl	C57BL/6 J BomTac	C57BL/6 J OlaHsd
TEG	*R*-time (min)	Median	10	30.42	42.60	29.98	28.85	25.53	22.65	22.48	22.45	24.93
		%CV	10	33	28	54	30	38	31	18	17	14
		Median males	5	30.85	30.05	22.55	31.20	25.10	15.40	19.55	26.00	24.05
		Median female	5	22.70	50.00	37.40	27.85	25.95	28.45	23.40	21.90	28.65
	Maximum amplitude (mm)	Median	10	47.05	53.35	55.65	54.17	53.62	55.47	52.20	57.70	54.15
		%CV	10	14	11	7	7	12	18	6	14	8
		Median males	5	49.15	56.70	59.55	54.80	55.95	63.65	54.20	63.10	58.05
		Median female	5	44.95	47.65	55.30	53.55	47.00	45.95	48.65	48.65	52.20
Coagulation parameter	aPTT (s)	Median	10	31.15	38.85	41.80	34.70	33.25	35.40	38.00	37.80	35.55
		%CV	10	7	13	13	12	15	15	7	8	12
		Median males	5	30.60	37.00	34.40	32.70	28.50	33.70	37.50	37.40	34.70
		Median female	5	33.00	39.50	43.40	36.00	35.00	36.00	38.00	38.00	35.70
	PT (s)	Median	10	7.70	8.30	8.55	8.30	7.90	8.75	8.35	7.80	8.00
		%CV	10	2	3	6	2	12	7	6	6	9
		Median males	5	7.70	8.50	8.30	8.20	7.20	8.70	8.30	7.70	7.60
		Median female	5	7.60	8.30	8.70	8.30	8.70	8.90	8.50	8.20	8.80
	Fibrinogen (mg/dl)	Median	10	155.50	154.00	153.00	154.00	158.00	120.00	143.00	167.00	179.50
		%CV	10	19	38	15	13	41	31	10	32	35
		Median males	5	183.00	181.00	171.00	169.00	260.00	153.00	151.00	209.00	251.00
		Median female	5	124.00	145.00	142.00	136.00	116.00	109.00	127.00	135.00	131.00
Tail-tip Bleeding^a^	Blood loss (mg)	Median	20	23.50	10.50	13.50	19.00	11.50	469.50	64.50	36.00	348.00
		%CV	20	118	79	36	32	44	81	171	93	85
		Median males	10	27.00	0.00	12.00	18.00	10.00	869.50	93.50	27.50	764.50
		Median female	10	21.50	15.00	15.50	19.50	15.50	227.50	37.00	50.50	75.50

a Data derived from the study published by Schiviz et al.^6^

Generally, no marked differences were observed in median MA between strains or substrains (Table 1). Median MA was 47.05 mm (CV 14%) in 129S1/Sv mJ mice. Within-substrain and between-substrain variation in MA was relatively small among BALB/c substrains (CV range: 7–12%; median range: 53.35–55.65 mm).

Coefficients of variation in MA were comparably small within C57BL/6 strains, ranging from 6 to 18%. Median MA among C57BL/6 substrains varied similarly to BALB/c substrains, ranging from 52.20 to 57.70 mm. Male mice consistently showed a tendency toward greater MA across all strains and substrains, with up to a 1.4-fold difference in C57BL/6 J Crl (Table 1).

### Coagulation parameters and fibrinogen

Median aPTT was 31.15 s in 129S1/Sv mJ mice (CV 7%) (Table 1). Longer and more varying values for aPTT were observed for the BALB/c substrains. Within-substrain and between-substrain variation was relatively small in aPTT among BALB/c substrains (CV range: 12–15%). The median range for aPTT among BALB/c substrains varied from 33.25 s (BALB/c J) to 41.8 s (BALB/c OlaHsd) (Table 1 and supplementary Figure S1(a)).

Coefficients of variation in aPTT within C57BL/6 strains ranged from 7 to 15%, while median aPTT among C57BL/6 substrains ranged from 35.40 s (C57BL/6 JCrl) to 38.00 s (C57BL/6 NCrl). Males generally showed a shorter median aPTT than females (supplementary Figure S1(b)).

Median PT was 7.70 s in 129S1/Sv mJ mice (CV 2%) (Table 1). Longer and more varying values were seen in other substrains for median PT, ranging from 7.80 to 8.75 s in BALB/c and C57BL/6 substrains (CV range: 3–12%), and thus did not reveal differentiated coagulation in the substrains. BALB/c J Crl males had significantly shorter PT than females (data not shown), whereas no sex differences were observed in other substrains (Table 1).

**Figure 2.**
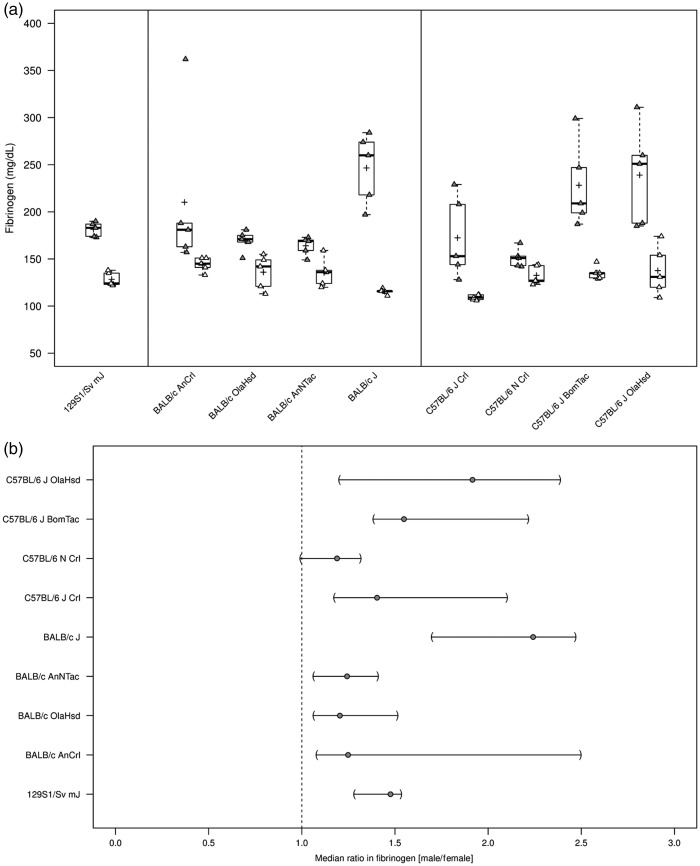
Plasma fibrinogen levels (mg/dl) in male and female wild-type 129S1/Sv mJ, BALB/c, and C57BL/6 substrains. (a) Plasma fibrinogen levels (mg/dl) summarized graphically by substrain and sex using boxplots (grey = males; white = females; see Figure 1(a) for a detailed description of boxplots). (b) The median ratio in fibrinogen between sexes per substrain and corresponding two-sided 95% CIs (see Figure1(b) for a detailed description of interpretation of CIs).

Median plasma concentration of fibrinogen across all substrains ranged from 120.0 mg/dl in C57BL/6 JCrl to 179.5 mg/dl in C57BL/6 JOlaHsd mice (Table 1). With the possible exception of C57BL/6 N Crl substrain (CV 10%), the variation in plasma fibrinogen levels was notable in all substrains (129S1, BALB/c, C57BL/6), ranging from 13 to 41%. This variation was due to a pronounced sex difference for plasma fibrinogen levels, with consistently greater values in males than in females in all substrains (Figure 2(a)). Median fibrinogen ratios (male/female) were 1.5-fold in 129S1/Sv mJ and most pronounced in BALB/c J (2.2-fold) and C57BL/6 OlaHsd (1.9-fold) substrains (Figure 2(b)).

### Tail-tip bleeding assay (blood loss)

Median blood loss (mg) and ranges thereof in the 129S1/Sv, BALB/C, and C57BL/6 substrains as recorded in a tail-tip bleeding assay by Schiviz et al^6^ are provided in Table 1. The data demonstrate relatively lower blood loss in 129S1/Sv mJ and BALB/c substrains (median range: 10.5–23.5 mg) v. relatively higher blood loss in C57BL/6 substrains (median range; 36–469.5 mg), with substantial variability in C57BL/6 JCrl and C57BL/6 J OlaHsd substrains (supplementary material, Figure S2(a)). Additional analyses of both C57BL/6 substrains confirm marked sex differences in blood loss (mg) with significantly higher blood loss in male mice and median ratios (male/female) of up to 10.1-fold (supplementary material, Figure S2(b)). Conversely, while overall blood loss (mg) was substantially reduced in all wild-type 129S1/Sv mJ and BALB/c substrains, sex differences suggest greater blood loss in female BALB/c mice (supplementary material, Figure S2(b)).

## Discussion

Haemophilic mouse models are the most commonly used animal models to test the primary pharmacodynamics of products to treat haemophilia. Such studies are frequently designed to show a correction of the haemophilic phenotype in genetically modified mice, often by comparison with wild-type mice. However, as different wild-type mouse strains and haemophilic mice on different genetic backgrounds show different bleeding phenotypes,^2-6^ it is critical that the genetic background of the haemophilic mice resembles that of their comparators.

A study assessing the bleeding phenotypes of various FVIII knock-out (and wild-type) mouse strains in the tail-tip bleeding model revealed marked differences not only between mouse strains, but between substrains and between the sexes.^6^ Haemophilia A mice (FVIII^−/−^) on a BALB/c background showed a milder haemophilic phenotype (blood loss) than FVIII^−/−^ mice on a C57BL/6 background. However, bleeds in wild-type mice also showed marked differences among strains and substrains of the same strain, as well as between males and females. Some substrains of C57BL/6 wild-type mice had blood loss reminiscent of a haemophilic phenotype and with high inter-animal variability. BALB/c and 129S1/Sv wild-type mice showed low median blood loss and reduced variability.^6^ These phenotypes were inherent to the substrains despite earlier efforts to standardize the model and to reduce inter-animal variability.^13–15^

These findings prompted us to comprehensively characterize the coagulation phenotype of three strains of wild-type mice on different genetic backgrounds using *ex-vivo* assays, with less interference from environmental or (patho-)physiologic parameters not directly linked to coagulation (e.g. room and body temperature, heart rate, blood pressure, response to anaesthesia) than in *in-vivo* tests.

We characterized the phenotype of 129S1/Sv mJ, BALB/c and C57BL/6 substrains applying TEG, coagulation parameters (aPTT, PT) and plasma fibrinogen, which are well-accepted *ex-vivo* endpoints for studying efficacy in the treatment of haemophilia and other blood clotting disorders.

TEG showed higher within-substrain and between-substrain variation in *R*-time in BALB/c substrains than in C57BL/6 substrains. Marked sex differences in *R*-time, however, were detected in particular substrains of both strains (i.e. BALB/c AnCrl, C57BL6/ J Crl, C57BL6/ N Crl). It is noteworthy that *R*-time variations could also naturally result from various levels of activation of intrinsic system. We therefore applied a TEG assay system that is in routine use in our laboratory and had been carefully validated in previous studies.^10^ Other TEG parameters (i.e. alpha-angle and *K*-time) were not included in the current study but should be considered by investigators in establishing particular wild-type substrains as controls.

Several physiological parameters have been described that are affected by substrain differences and these also change with age.^16–18^ For example, platelet counts in male C57BL/6 J mice were shown to rise from 1156 platelets/µl at 6 months of age to 2133 platelets/µl at 12 months of age, while those in female C57BL/6 J mice remained stable (1268 and 1229 platelets/µl). In contrast, other strains (e.g. 129S1/sv InJ and BALB/c ByJ) showed no major changes in platelet count with age.^6,19^

Sex differences in coagulation have been reported in laboratory animals as well as in humans, influencing clinical practice.^20-22^ In mice, our findings are in accordance with previous studies showing sex differences in aPTT and fibrinogen,^23,24^ with female mice of particular substrains having longer *R*-time (BALB/c AnCrl, BALB/c OlaHsd, C57BL6 J Crl, C57BL6/ N Crl, C57BL6 OlaHsd), a tendency (with varying confidence) to longer aPTT, and consistently lower plasma fibrinogen levels than males in all substrains investigated. It has been reported that ovariectomized mice showed similar coagulation values to those in male mice,^24^ highlighting the role of steroid sex hormones in modulating the coagulation cascade.^25^

It is generally recognized that different inbred wild-type mouse strains show distinct phenotypes, which influence body weight and size, behaviour and (patho-) physiologic processes.^18^ As these differences even include organ morphology,^26^ it was perhaps not surprising that the genetic background affected coagulation and susceptibility to coagulation disorders.^23,27^

An example of the effect of genetic diversity relevant to the present study is a spontaneous mutation in C57BL/6 JOlaHsd mice (deletion of multimerin 1 and α-synuclein) that causes thrombus instability and impaired platelet function.^28^ This finding is in line with the high blood loss observed in the tail-tip bleeding assay.^6^

In contrast to the tail-tip bleeding results, where blood loss in certain wild-type mice of C57BL/6 substrains (J Crl, JOlaHsd) was particularly pronounced,^6^ *ex-vivo* data demonstrated no marked coagulation differences between 129S1/Sv mJ, BALB/c and C57BL/6 strains.

While C57BL/6 JOlaHsd mice showed a prominent bleeding phenotype in wild-type males, *ex-vivo* TEG and coagulation parameters (aPTT) were mid-range, and albeit more variable, comparable with other strains, and fibrinogen levels were similar to or higher than in other strains. MA, a parameter for clot strength which is heavily influenced by platelet function,^29^ was also mid-range but significantly higher in C57BL/6 JOlaHsd males than females. This sex difference in MA was generally observed for other C57BL6 substrains.

In addition, female wild-type mice in C57BL/6 substrains (J Crl, J OlaHsd) tended to have longer *ex-vivo* clotting time (*R*-time), reduced MA, and prolonged aPTT; however, females had significantly less blood loss in the tail-tip bleeding model than male mice of the same C57BL/6 substrain. While the underlying mechanism for these observations is unknown, an influence of hormonal status has been suggested previously.^24,25^

These substrain/sex differences are an important aspect of this study and indicate that isolated *ex-vivo* analyses do not completely mirror the pathophysiological mechanisms in a living mammal following injury and blood loss. *Ex-vivo* analyses, however, often did correlate well with each other in describing the *ex-vivo* coagulation phenotypes. As one example, TEG results (i.e. relatively reduced *R*-time) in BALB/c J mice were reflected in other coagulation variables, showing shorter median aPTT and median PT than other BALB/c substrains. Thus, coagulation data from analyses of *ex-vivo* parameters appeared congruent within substrains.

On the other hand, while blood loss in the tail-tip bleeding assay is relatively reduced in wild-type BALB/c strains,^6^ our analysis indicates that female mice of BALB/c substrains (AnCrl, J, and OlaHsd) bled markedly more than males of the same substrain. This difference appeared to be reflected *ex vivo* by a tendency toward longer aPTT or PT, reduced MA and fibrinogen levels in samples from female mice of BALB/c substrains (AnCrl, J and OlaHsd).

Based on our results, it appears that an ideal mouse strain for all preclinical efficacy models for testing of haemophilia compounds has not yet been identified. Depending on the model used and the parameters assessed, results between strains or substrains of the same strain, as well as between the sexes, may vary considerably. It is worthwhile, however, to identify the optimal strain for the experimental models used, as, for example, major sex differences can lead to high overall inter-animal and group variabilities.

The use of one sex only might be considered during preclinical study design. However, this has to be carefully aligned with the intended target clinical patient population and undergo critical ethical considerations related to laboratory animal breeding and use, as roughly 50% of purpose bred animals are not used. Furthermore, it is imperative that laboratories establish their own normal values for their assays and animals.^30^

A technical limitation of the current study is the large numbers of substrains and endpoints that are currently considered for studies in haemophilia mouse models by various investigators in the field. Nevertheless, our comprehensive *ex-vivo* results lead us to suggest using whenever possible similar strains/substrains as controls matched to the haemophilia model selected.

Comparison of blood loss in knock-out mice with different backgrounds showed a marked reduction in inter-animal variability by back-crossing in the preferred substrain,^6^ which, if confirmed in future studies, may allow a reduction in animal number per group. In keeping with the animal welfare practice by *replacement, reduction*, and/or *refinement* (3Rs), we therefore conclude that it is crucial to align the mouse strain and/or backcrossing strategy of knock-out mice with the preclinical efficacy model used.

## Supplemental Material

Supplemental material for Coagulation phenotype of wild-type mice on different genetic backgrounds Click here for additional data file. Supplemental Material for Coagulation phenotype of wild-type mice on different genetic backgrounds by Alexandra Kopić, Karima Benamara, Maria Schuster, Peter Leidenmühler, Alexander Bauer, Helmut Glantschnig and Werner Höllriegl in Laboratory Animals
