# Supplementary material for: Coagulation phenotype of wild-type mice on different genetic backgrounds
Source: Lab Anim. 2018 Nov 12;53(1):43–52. doi: 10.1177/0023677218811059 (PMC6416704; doi:10.1177/0023677218811059)
Supplement: Supplemental material for Coagulation phenotype of wild-type mice on different genetic backgrounds [file Supplemental_Material.pdf]

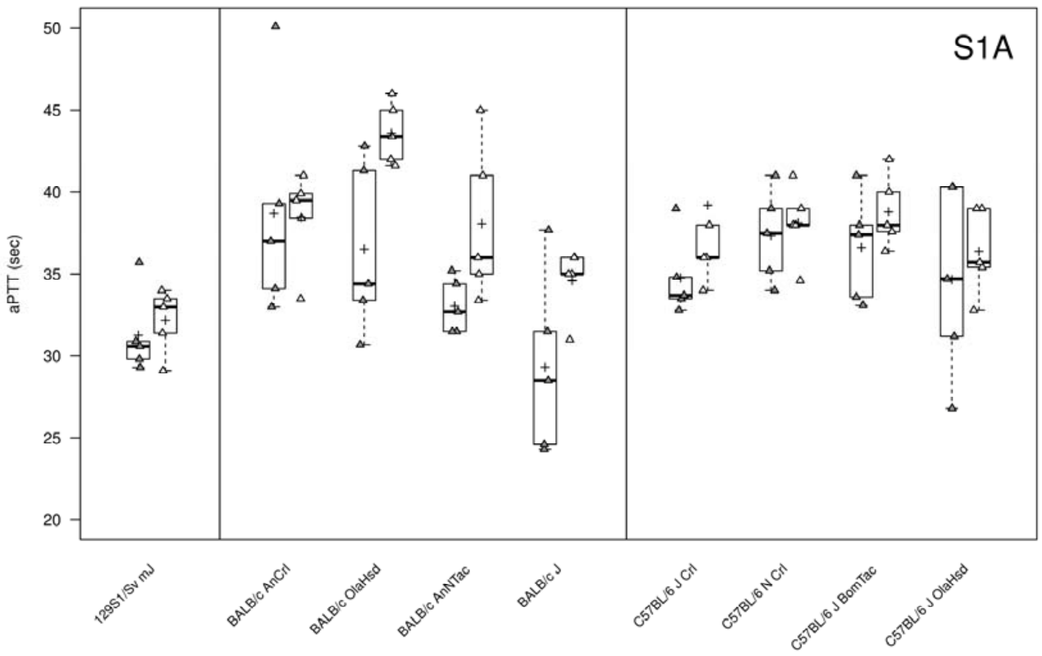
S1A
aPTT (sec)
50
45
40
35
30
25
20
129S1/Sv mJ
BALB/c AnCrl
BALB/c OlaHsd
BALB/c AnNTac
BALB/c J
C57BL/6 J Crl
C57BL/6 N Crl
C57BL/6 J BomTac
C57BL/6 J OlaHsd

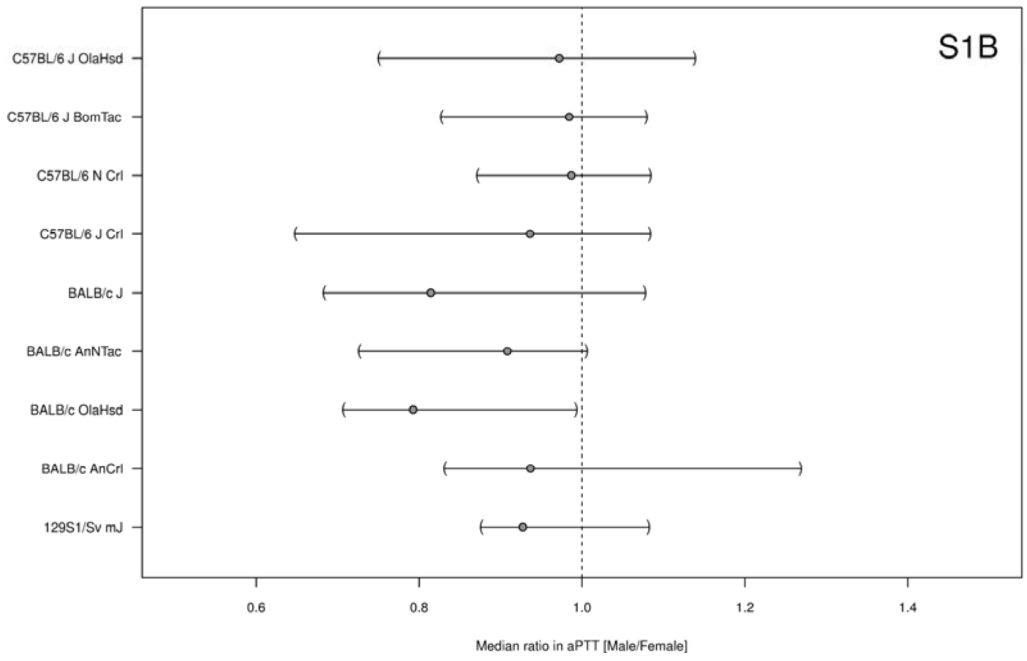
S1B
C57BL/6 J OlaHsd
C57BL/6 J BomTac
C57BL/6 N Crl
C57BL/6 J Crl
BALB/c J
BALB/c AnNTac
BALB/c OlaHsd
BALB/c AnCrl
129S1/Sv mJ
0.6
0.8
1.0
1.2
1.4
Median ratio in aPTT [Male/Female]

Supplemental Figure S1. Activated partial thromboplastin time (sec) in male and female wild-type 129S1/Sv mJ, BALB/c, and C57BL/6 substrains. Figure **S1A** shows aPTT summarized graphically by substrain and sex using boxplots (grey = males; white = females). Boxplots: The lower edge of the box represents the 25th percentile (or 1st quartile), the upper edge of the box represents the 75th percentile (or 3rd quartile), and the line within the lower edge and the upper edge of the box indicates the median. The distance from the lower edge to the upper edge of the box represents the inter-quartile range (IQR). A whisker is drawn above the 75th percentile to the largest data value that is less or equal to the value that is 1.5*IQR above the 75th percentile. A whisker is drawn below the 25th percentile to the smallest data value that is less or equal to the value that is 1.5*IQR below the 25th percentile. The cross represents the arithmetic mean. Individual measurements were added to the boxplots, where the exact horizontal position of plotting symbols was randomly determined. Figure **S1B** shows the median ratio in aPTT between sexes per substrain and corresponding two-sided 95% CIs. A two-sided 95% CI for the ratio not containing the value 1 is equivalent to rejecting the null hypothesis of no difference against the two-sided alternative at the 5% level of statistical significance. Two-sided 95% CIs should be interpreted with caution due to the small sample size per substrain and sex.

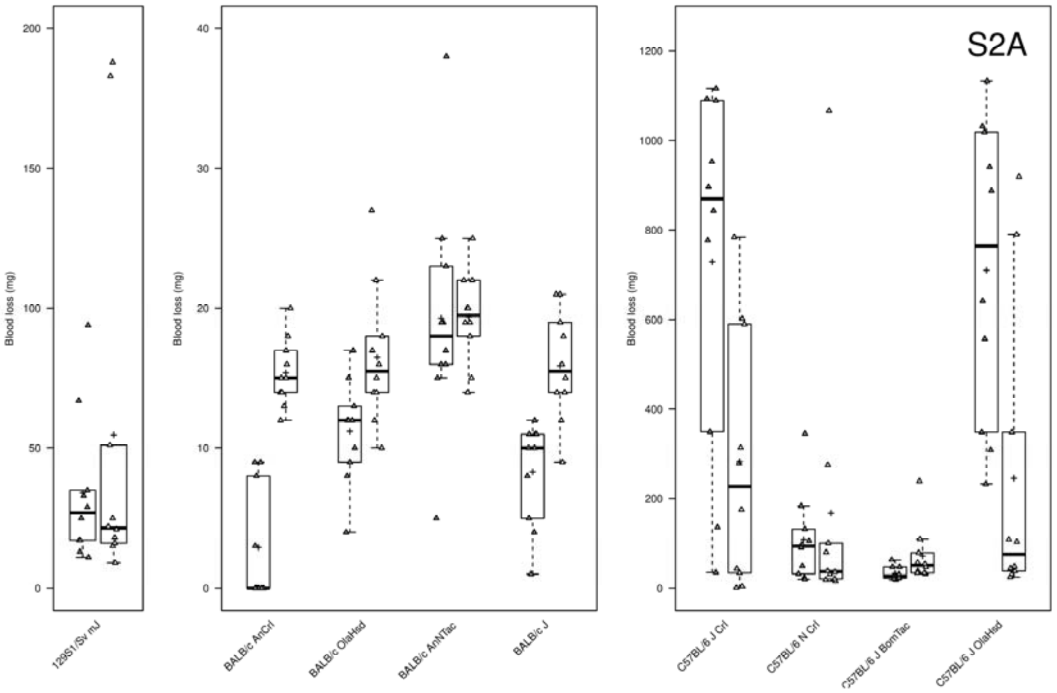
S2A
Blood loss (mg)
200
150
100
50
0
40
30
20
10
0
1200
1000
800
600
400
200
0
129S1/Sv mJ
BALB/c AnCrl
BALB/c OlaHsd
BALB/c AnNTac
BALB/c J
C57BL/6 J Crl
C57BL/6 N Crl
C57BL/6 J BomTac
C57BL/6 J OlaHsd

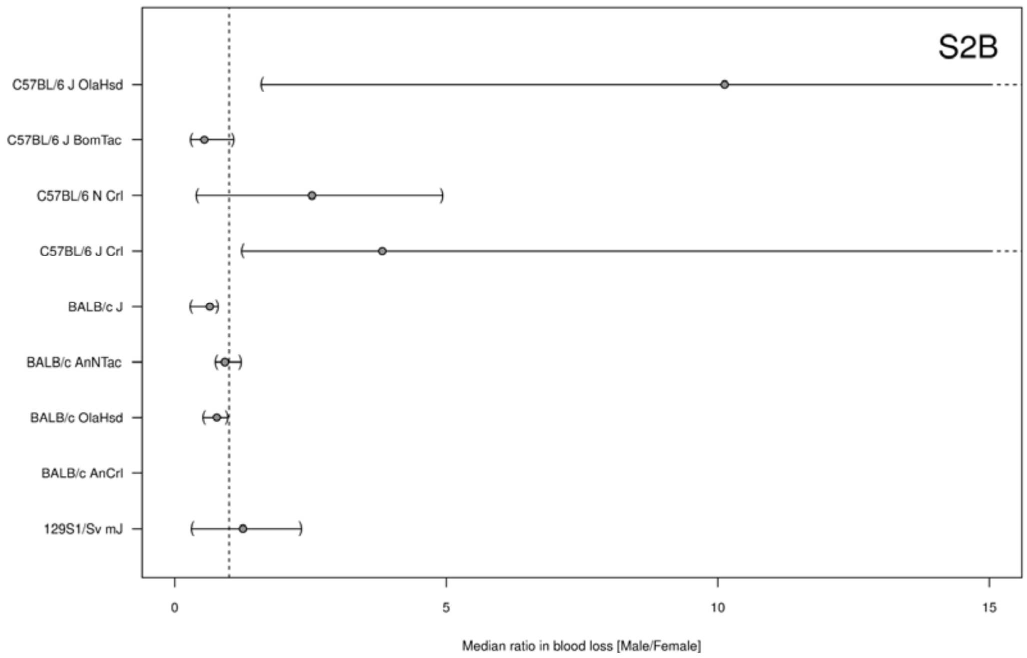
S2B
C57BL/6 J OlaHsd
C57BL/6 J BomTac
C57BL/6 N Crl
C57BL/6 J Crl
BALB/c J
BALB/c AnNTac
BALB/c OlaHsd
BALB/c AnCrl
129S1/Sv mJ
0
5
10
15
Median ratio in blood loss [Male/Female]

Supplemental Figure S2. Tail-tip bleeding model. Blood loss (mg) in male and female wild-type 129S1/Sv mJ, BALB/c, and C57BL/6 substrains. Figure **S2A** shows blood loss summarized graphically by substrain and sex using boxplots (grey = males; white = females; Refer to Fig.S1A for a detailed description of boxplots) and strain-specific y-axes. Note the scaling of y-axes to accommodate different bleeding phenotypes in mg blood loss. Blood loss was pronounced in particular C57BL6 substrains. Figure **S2B** shows the median ratio in blood loss between sexes per substrain and corresponding two-sided 95% CIs (upper limits were truncated). Refer to Fig.S1B for a detailed description of interpretation of CIs). For BALB/c AnCrl, no ratio was calculated as the median blood loss in males is zero.
